# Online selection of a physician by patients: the impression formation perspective

**DOI:** 10.1186/s12911-022-01936-0

**Published:** 2022-07-25

**Authors:** Zhengwei Huang, Chen Duan, Yanni Yang, Ribesh Khanal

**Affiliations:** 1 College of Economics and Management, China Three Gorges University, Number 8, Daxue Road, Xiling District, Hubei, China; 2 School of Literature and Media, China Three Gorges University, Hubei, China

**Keywords:** Online health communities, Impression formation, The Toulmin’s model of argumentation, Professional capital, Consultation intention

## Abstract

**Background:**

With the rapid development of online health communities (OHCs), an increasing number of physicians provide services in OHCs that enable patients to consult online in China. However, it is difficult for patients to figure out the professional level of doctors before consultation and diagnosis because of information asymmetry. A wealth of information about physicians is displayed in their profiles as a new way to help patients evaluate and select quickly and accurately.

**Objective:**

This research explores how the profile information (PI) presented in OHCs influences patients' impression formation, especially the perception of professional capital (i.e., status capital and decisional capital). The impression influences their intention to consult further, which is partially mediated by the initial trust. The Toulmin’s model of argumentation is used to decide the strength of the argument presented in physicians’ homepage information and divide it into claim, data, and backing.

**Methods:**

This study conducts an internet experiment and recruits 386 subjects through the internet to investigate the effect of impression formation on online selection behavior by a patient.

**Results:**

The results show that the strength of argument has a significant positive association with the perception of professional capital. Perceptions of professional capital are highest when a fully composed argument (claim/data/backing) is included in a profile, with claim/data being the next highest and claim only the lowest. Recommendations from connections have the strongest impact. In turn, patients' selection decisions are influenced by their perception of professional capital, which is partially mediated by initial trust.

**Conclusions:**

This study is significant in terms of its implications for theory and practice. On the one hand, this research contributes to the online health community literature and suggests that the perception of professional capital on physicians should be pre-presumed and built based on the information before in-person interaction online. On the other hand, this study is helpful in understanding the effect of various components included in PI on perceiving physicians’ abilities, and not all information is equally important.

**Supplementary Information:**

The online version contains supplementary material available at 10.1186/s12911-022-01936-0.

## Background

OHCs are prospering and have significantly aided patients in gaining access to medical consultation services regardless of time and space limitations. In 2020, the outbreak of the corona-virus epidemic increased demand for online diagnosis and consultation, as well as promoted the upgrading of services demand in the online medical community. According to China Internet Network Information Center (CNNIC), as of December 2021, there were 298 million online medical users.^1^ A representative type of OHCs is the patient-to-doctor communities, such as “Hao Daifu online” (https://www.haodf.com), “Chunyu Doctor” (https://www.chunyuyisheng.com/) and “Jiankang Net” (https://www.120.net). These websites provide opportunities for communication between doctors and patients [1].

Various research has found that physicians’ participation in OHCs could be motivated by extrinsic rewards, such as economic or social gains, or intrinsic rewards, such as varying levels of altruism [2–4]. Thus, it is not surprising that an increasing number of physicians are participating in OHCs to provide professional assistance in meeting patients’ healthcare needs. Existing research has shown that patients find it difficult to assess the quality of medical services before purchasing and consuming them due to information asymmetry between healthcare providers and patients [5]. One challenge in virtual communities is the elimination of face-to-face meetings, which increases uncertainty risk. To avoid the uncertainty risk, one potential solution is the physician’s personal homepage in OHCs, which help patients judge the professional capability of a physician and build trust.

**Fig. 1:**
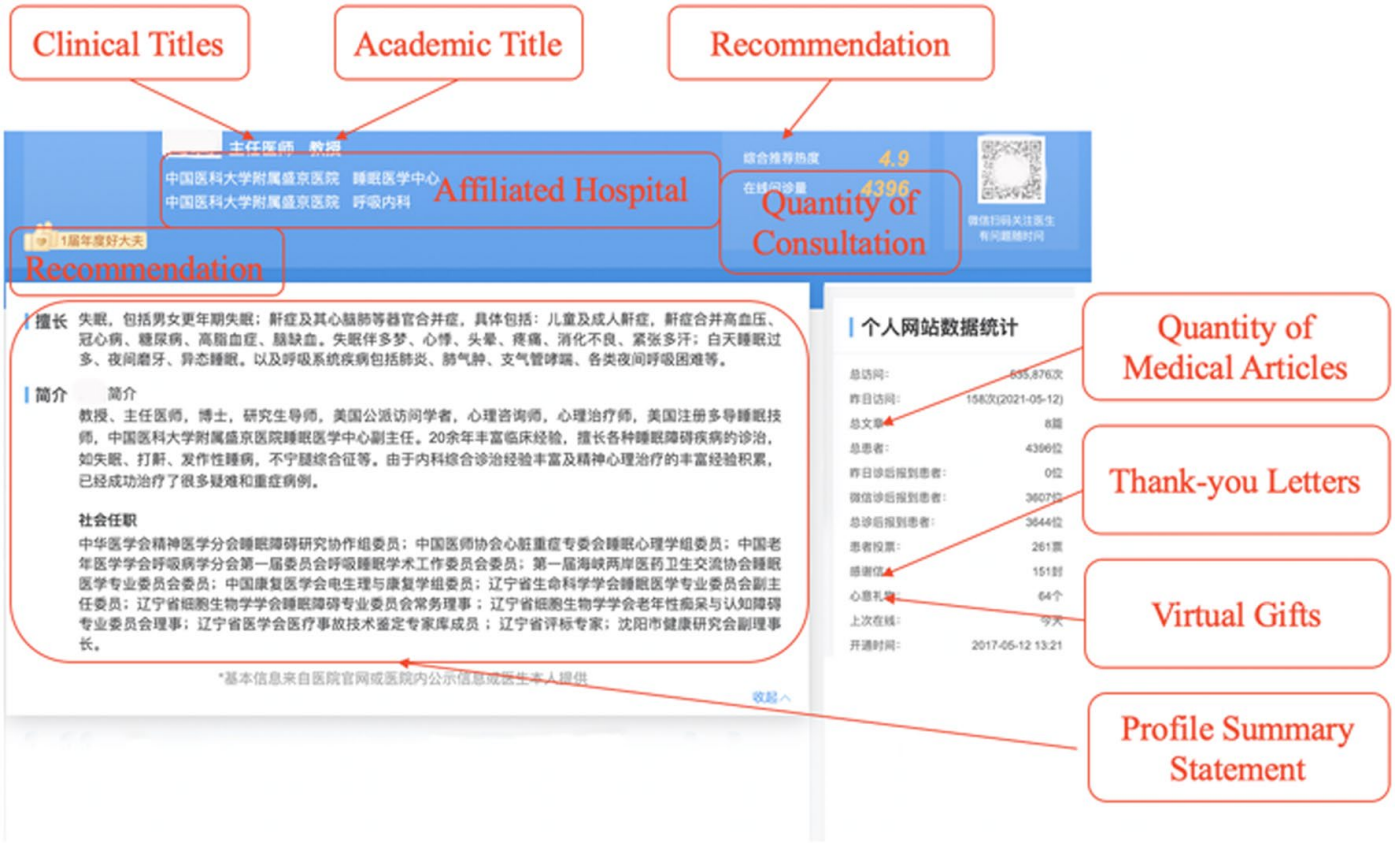
An example of a physician’s PI

For many patients, these websites have supplanted traditional in-person methods of interaction between physicians and patients to form a first impression of these physicians prior to the health consultation. On these websites, PI enables physicians to credibly convey their personal information about their service quality and competence level to everyone who visits these online sites, including a text (e.g., description of work history) or images (e.g., profile picture). In turn, patients can examine these online pieces of information presented to form the perception of clinical outcome and professional capability, and identify a high-expertise doctor to consult (Fig. 1) [5, 6]. In other words, the informational value indicates that high-expertise doctors can separate themselves from low-expertise doctors [7]. Thus, understanding how the information presented in the physicians’ PI influences online selection by patients is important.

Some researchers have long recognized the trichotomy of products and services’ search, experience, and credence qualities [8]. Consumers can easily decide the quality of search and experience goods after purchasing them. In contrast, the quality of credence goods cannot be determined even after purchase and direct personal experience with the product [13]. Healthcare services, in general, have credibility characteristics; however, these goods suffer from the worst information failures. Previous research has shown that PI of experience service is effective. For example, Joseph P demonstrates that the potential interaction outcomes between search and experience goods and user’s PI are not the same as that of credence goods [9]. Besides, prior research on search and experience goods has found that PI to be effective [10], but does this insight also extend to credence goods? Our paper primarily focuses on the role of PI in credence goods with chronic disease care, which has received little attention.

Specifically, the objective of our research is to investigate how a physician’s PI in OHCs affects patients’ online selection of a physician. The main research questions are as follows:

1. How does physician’s PI in OHCs affect patient selection online?
2. Is this information of equal value to patients?
3. How does the perception of physicians’ professional capital mediate the effects of physician’s PI on patients’ online decisions?

The contributions of this paper are threefold. First, our research extends the research and theory to understand how patients interpret physicians’ PI. Our study illustrates that physicians’ PI in OHCs can signal for patients to separate high-expertise doctors from low-expertise doctors. Results suggest that patients take full advantage of the various information included in the profile to form the perception of physicians’ professional capability prior to the interaction in OHCs. Second, the result provides evidence that not all information has equal value; the information generated by the third party has a greater impact than self-generated information. Third, this paper investigates the mediating effects of initial trust on the relationship between the perception of professional capital and patients’ online decisions.

The rest of this paper is structured as follows. In “Literature review” section, we critically review of relevant literature and theories. “Research model and hypotheses” section then presents the research model and hypotheses. In “Methods” section, we describe the research procedure, data collection methods, and variable measurement. Finally, we present and discuss our results, conclusions, and implications for future research.

## Literature review

### Online health communities

OHCs offer a greater variety of information about physicians than traditional medical settings like hospitals and enable doctors to better help and serve patients by performing various functions [11, 12]. OHCs help doctors and patients because doctors can use these functions to achieve their goals more efficiently, while patients can search for health information and suggestions that can help them recuperate faster and more effectively. Several studies have researched the benefits of OHCs from different perspectives. On the one hand, doctors can not only earn a higher income (including virtual gifts and extra bonuses) but also accumulate rich clinical experience through these internet platforms. In addition, they improve their online and offline reputations to meet their self-fulfillment needs (self-respect and being well-respected) [2]. On the other hand, patients can access more professional medical information or diagnosis without the limitation of time and space [13], which significantly reduces the cost of outpatient visits and further enhances their health management awareness. Besides, they can freely select a suitable physician who can help them recuperate faster and more effectively according to the affiliated institutions and departments in the virtual environment. It improves the efficiency and efficacy of diagnosis and treatment and decreases doctor-patient conflict [14].

In OHCs, multiple information displayed on the physicians’ homepage has a significant influence on patients’ decisions [15], and not all information has equal value [16]. Some studies confirmed the effect of patient-generated content (such as electronic word-of-mouth, digital gifts and thank-you letter), system-generated information (like contribution-value) [14] and physician-generated information [17] on patients’ behavior and decision-making at different stages. The electronic word-of-mouth shown on the physicians’ homepage is an important source of information relied upon by patients during the decision-making process [18]. User-generated and system-generated information has attracted considerable research in the past decade [19–22]. However, as credence goods, medical service, is widely viewed as a professional service rather than a simple business process. Physicians have more information than patients. Therefore, the role of physician-generated information becomes important in this specific healthcare context. There is little research on how physician-generated information influences patients’ selection in OHCs.

Although PI on the websites is of great significance for patients’ online consultation intentions, the researches on the OHCs has primarily focused on the factors influencing information sharing behavior [23], health information seeking on the ongoing interactive online [24, 25], information adoption at the decision-making stage [26], service satisfaction and medical team performance [27]. There has been little research exploring how PI on the online communities’ influences patients’ consultation intention during information seeking stage, and no one has considered the effects of the strength of argument presented in these pieces of information on professional level and social resource perception.

The prior literature on the use of information technology (IT) and user-generated content (UGC) in healthcare communities has mainly examined the potential impact of demand-side physician reviews (such as online word-of-mouth and online rating) on the patient recommendation and patients’ online decision-making when selecting doctors [28–30]. However, the impact of doctors’ PI has not been well understood from the supply side. As a result, this research investigates the impact of doctor-provided information on online patient selection. Our research adds to this literature and provides new empirical evidence in this critical area.

### Impression formation

Originated from social psychology, impression formation illustrates that when individuals primarily met a stranger or formed relationship in the early stage, they make judgments based on limited information on some attributes or overall characteristics of cognitive objects, such as personality traits, professional skills, and social class. The first impression will have an anchoring effect on individuals, influencing future observations and interactions [27]. The impression formed during the first stage does not significantly change over time and lays a fundamental basis for the project’s success and decision-making. Previous studies have shown that impression formation will significantly influence the trust and preference of the cooperative partners [31, 32]. Online health websites, rather than face-to-face consultations, change the way patients form their first impressions of physicians. As a result, it is critical to comprehend the impact of impression formation on consultation intention.

Traditionally, people form their first impression of others through an initial face-to-face encounter. From these direct encounters, individuals interpret “cues” into attributes of the person [33]. Online health websites, rather than face-to-face consultations, change the way patients form their first impressions of physicians because they provide more information than the traditional method. The first impression can be formed in the online environment based on digital materials, such as personal PI, portrait, video, and website design, without face-to-face interaction. First and foremost, when users hear a new voice, they will automatically form an initial impression of the personality of the voice owner [34]. Second, previous studies have shown that the users’ photos [35], age, and gender [31] will produce the differences in the formation of initial impressions and profoundly influence the subsequent behavior of users [36]. In social networking sites, design elements of the website page, such as layout and color, have an effect on the impression formation of other users and preferences [37]. Users can examine others' characteristics through online search information "clues" which may be text(target words, health condition, social activities, and work experiment) or image(photo, portrait) [38], and explore the strategy of self-impression management further. Existing studies discussed the initial impression formation from the two aspects: the information presentation format and the inherent material properties. However, based on the Toulmin’s model of argumentation, this paper understands how impression formation in online communities influences the perception of these physicians.

### The Toulmin’s model of argumentation

As one of the most enduring theoretical models of argument, the Toulmin's Model of Argumentation can effectively examine the strength of an information argument and has been used to research information presented in the online environment [7, 39], which focuses more on the application of logic in human situations [40]. Based on the Toulmin’s model of argument, the strength and persuasiveness of an argument are determined by specific elements such as claim, data, and backing [7]. Claims refer to the self-generated conclusion that an individual asks the reader to believe. Data that directly supports the claim strengthens the argument, including added facts and evidence. Backing supplies, the evidence or support that the data is true and should be accepted. The strength of an argument increases with the addition of the above elements so that an argument with claim, data, and backing is more convincingly supported than an argument with claim and data, which is stronger than an argument with claim only.

**Fig. 2:**
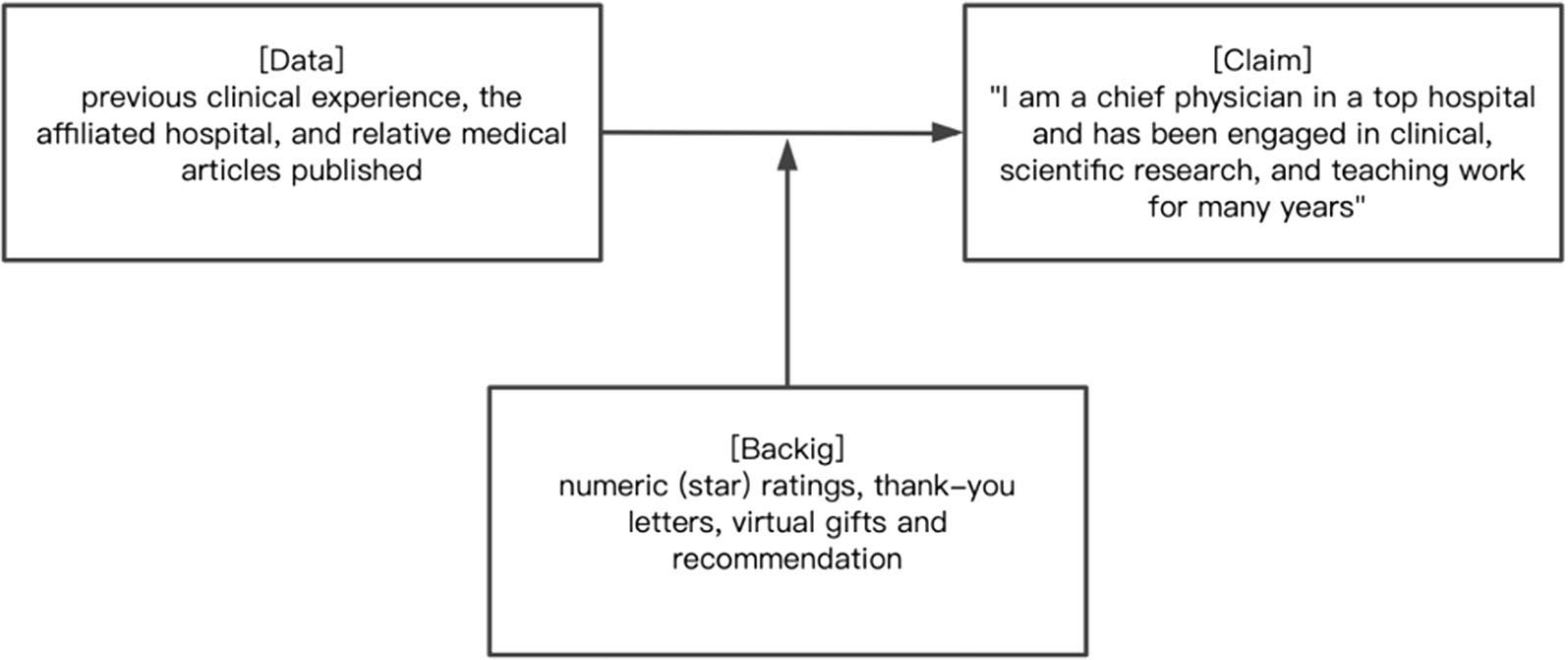
The Toulmin’s model of argumentation—sample argument in OHCs

In OHCs, a doctor claims that he or she is a chief physician of a prestigious hospital and claims they have been engaged in clinical, scientific research, and teaching work for many years. Nevertheless, trusting what the physician says based on a claim is insufficient; additional supporting data such as previous clinical experience, an affiliated hospital, and relative medical articles that are partly self-generated, should be provided. However, evidence suggests that individuals are more likely to exaggerate their achievement and progress in the online environment [41].The initial perception to a doctor entirely depends on a secondhand impression, whose accuracy is still questioned by many scholars [42]. Even if there is supporting data, patients may question whether these contents are physicians’ reliable indicators of Clinical Outcomes and expertise, because the claim and data are all subjective information provided by the physician and may be subject to self-manipulation. PI in OHCs can be self-generated or system-generated [14]. To improve patients' perceptions of expertise, additional backing is introduced to provide a system-generated endorsement for the claim and data, as shown in Fig. 2. Hence, patients are willing to choose and consult.

Little attention has been given to Toulmin’s model in influencing consumer belief on the Internet. Dongmin Kim indicates that an argument's strength increases consumers' trust in the context of an e-commerce setting [39]. Jeff Cummings examines how the strength of the argument presented in a firm’s enterprise social networking site (ESNS) impacts the future team members’ perceptions of social capital [7]. In addition, Ye and Johnson (1995) used Toulmin’s model to the develop explanations used in expert systems in an experimental setting, and empirical results show that explanations that come from Toulmin’s model are more persuasive in convincing users to accept an expert system’s conclusions than those that do not [43]. There has been very little research effort devoted to investigating whether arguments presented in the physicians’ PI actually increase patients’ perception of professional capital and, more importantly, on how to increase their impact on impression formation and building initial trust. To address this gap, this paper argues that the physicians’ PI influences patients' impressions of the profile owner’s professional capital, influencing their initial trust and consultation intention in OHCs.

### Professional capital

Professional capital is the renewable and valuable social capital developed through good education and associated with social professionals such as doctors, teachers, and lawyers [44] because it relates to power advantage and professional commitment. In doctor-patient communication, the professional capital acts as an exchange resource that reflects their status in the social structure and decision-making behavior. These resources are viewed as a potential identity symbol or the ability to dispatch and use resources through decision-making behavior. As a result, the professional capital can be divided into status capital and decisional capital [44, 45].

First, status capital, unrelated to the doctor's online behavior, stands for the individual and social advantages in society and is a structural strength with official certifications (e.g., education level, job title, affiliations, etc.) that can help other participants assess the personal and social advantages of social professionals and make decisions about social interactions. In OHCs, the status capital of a doctor should be determined by the social status of the doctor, that is, by his or her academic title (e.g., professor or associate professor), clinical title (e.g., chief physician, associate chief physician, resident physician, assistant physician), and different hospital ranking and geographical differences. In general, physicians with more senior titles and positions are given higher priority and privileges, and the higher the hospital level to which they belong, the more resources they have. Therefore, doctors use PI not only to introduce themselves but also to signal their status or the use of resources through decision-making. This strategy is consistent with the signaling literature in game theory [46, 47]. In conclusion, status capital, as one dimension of professional capital, is defined as the personal and social advantages of the structural power of social professionals. Gaining higher status capital is a long-term goal that evolves gradually over time [2].

Decisional capital, considered a decision behavior, is driven by the ability and willingness to make precise medical treatment [48]. In contrast to status capital, physicians' decisional capital cannot be identified without the dynamic interaction between physicians and patients and could be translated into exchange behavior in online counseling [45]. Physicians who provide online counseling services must send "signals" to patients, such as providing more counseling services and publishing more online articles, in order to increase trust and steer patients to online counseling services [2]. Patients can evaluate the value of doctors in OHCs by the frequency and distribution of interactions such as the model and quantity of sharing, the channels of online and telephone consultations, and the number of medical articles. In this way, it reflects doctors' abilities and diligence. On the one hand, the abilities include doctors' expression, judgment, insight, and inspiration in complex situations, which indicates that a doctor has extensive clinical experience when diagnosing a patient's suffering condition accurately. On the other hand, a physician's willingness reflects his or her attitude toward interacting with patients in OHCs and demonstrates the commitment to social professionals. Consequently, as another dimension of professional capital, decisional capital is defined as the ability to make correct judgments and the commitment to social occupation, which is always the basis for patients’ choices.

## Research model and hypotheses

### Impression formation and professional capital

In institutionalized social structures, doctors with higher status capital tend to be more dependable and valuable. Higher professional titles show that doctors are officially considered better (e.g., expert, knowledgeable, etc.), and patients are more likely to choose such physicians because they can view their status such as physician titles, as the quality of their diagnosis or counseling. In addition, participants with strong social advantages in the social structure should have more and/or better options, which they can use to obtain more resources. Likewise, participants in a better organization tend to have greater control over resources accordingly. For instance, an excellent hospital is often associated with more qualified doctors, more advanced equipment, and even other quality hospitals. According to a relevant study, developed cities control more healthcare knowledge and resources. In other words, the organization (i.e., the hospital) and geographical location (i.e., the city) reflect the social advantage of doctors in terms of better resources. Patients may also bring positive benefits (social and economic returns) to doctors who supply better social advantages (such as higher hospital and city levels). As a result, this study believes that status capital, including the structural advantage of doctors' personal and social power, will aid in making the correct diagnosis and give corresponding medical advice. Thus, we hypothesize:

#### H1a

The physician’s PI composed of claim, data, and backing can result in higher perception of the status capital than those with claims and data.

#### H1b

The physician’s PI composed of claims and data can result in a higher perception of the status capital than those with claims only.

Like prior dimensions, decisional capital, considered a decision behavior, is driven by the ability and willingness to make precise medical treatment. Physicians' decisional capital cannot be found without the dynamic interaction between physicians and patients and could be translated into exchange behavior in online counseling. The decisional dimension self-generated claim is a self-generated statement within the profile summary that describes doctor-patient relationship and previous clinical experience. Data supporting this claim appears in PI, such as cumulative quantity of consultations online, quantity of medical articles published, and prior successful projects [2]. This may not directly translate to physicians’ reliable indicators of the quality of care, but it does suggest that the physician has rich clinical experience when accurately judging the suffering condition. However, this information is still self-generated, which has the potential to be manipulated or misrepresented by the profile owner [49]. The inclusion of information such as a system-generated recommendation can provide additional backing and verification that the profile owner does indeed have the experience presented in the data [50]. Thus, PI containing a claim, data, and backing will elicit higher perceptions of decisional capital than a profile consisting of a claim and data only. A profile with a claim and data will elicit higher perceptions than claim-only profiles. As a result, an increasing number of patients will come to seek medical advice. Meanwhile, increased interaction shows that doctors are more enthusiastic and dedicated. All the above analysis leads us to the following hypothesis:

#### H2a

The physician’s PI composed of claims, data, and backing can result in a higher perception of the decisional capital than those with claims and data.

#### H2b

The physician’s PI composed of claims and data can result in a higher perception of the decisional capital than those with claims only.

### Professional capital and initial trust

Trust is the relationship between people, organizations, and events that refers to one person's uncertainty and expectations about another person's future behavior and is easily influenced by the actions of others [38, 51]. It is critical to the study of online communities because it has a significant impact on consumer behavior [52]. According to the formation stage of trust, it can be divided into initial trust and cumulative trust [53]. The initial trust is established during the first interaction [39, 53], and occurs when the two parties are unfamiliar. As the user's interactions increase, his or her initial trust transforms into cumulative trust. Based on the original state of patient impression formation in OHCs, we discuss the initial trust generated in a brief time when patients browse the doctor's home page information without any prior experience, which is quite different from the accumulative trust. Previous theoretical studies on trust have shown that, although the initial trust is temporary, it still affects patients' medical choice behavior and subsequent interactions, which means that the overall trust is shaped in the context of the initial trust [52, 54]. Initial trust in OHCs is influenced by the information on the doctor's PI because patients who visit OHCs for the first time form their first impression of the doctor's professional ability based on the limited information they already know, and this memory influences patients' initial trust. So, our study hypothesizes:

#### H3a

In OHCs, physicians' status capital has positive effects on patients' initial trust.

#### H3b

In OHCs, physicians' decisional capital has positive effects on patients' initial trust.

### Initial trust and consultation intention

**Fig. 3:**
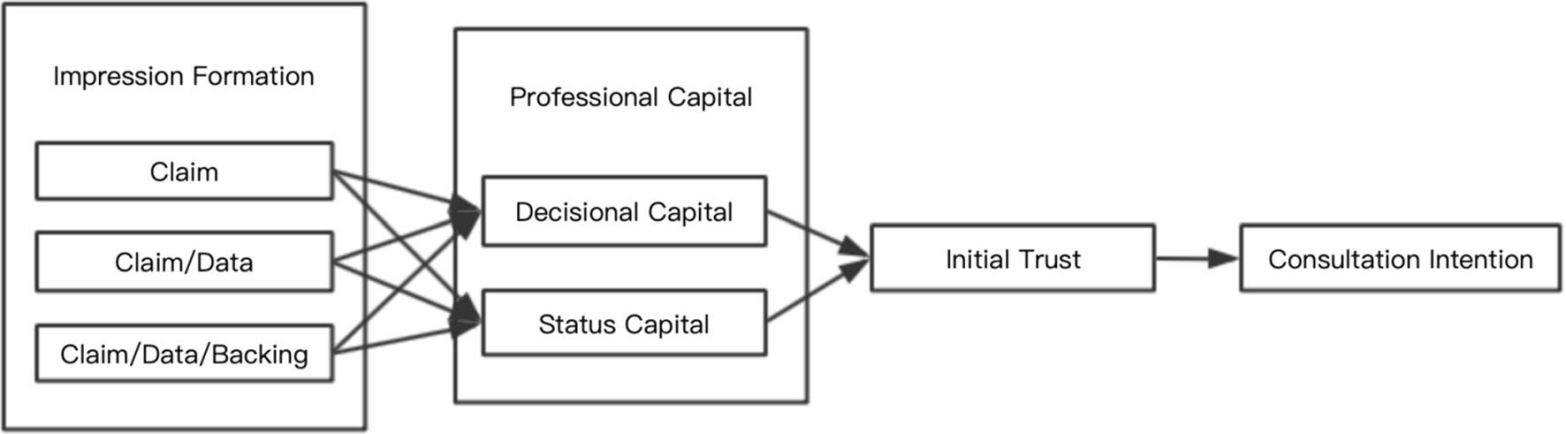
Conceptual model

Existing research has shown that when a new service is not well known to the public and involves uncertainty or potential risks, users usually decide whether to adopt it based on trust assessments. Initial trust is critical in this process because it eliminates perceived risk and uncertainty in interaction [45]. OHCs have higher uncertainty and risk than traditional medical services. Online patients actively seek doctors who can solve diseases in their minds through the internet and then look for medical consultation services, medical advice, and solutions. Given the influence of initial trust on the willingness of patients to choose their doctor for consultation [54], we construct the theoretical model as shown in Fig. 3 and proposed hypothesis 4:

#### H4

In OHCs, patients' initial trust has a positive impact on their consultation intentions.

## Methods

### Participants

Participants are from the university's online message board. The empirical data for this research was collected using an internet experiment with several advantages over traditional surveys, such as fast response time, cost-efficiency, and an absence of geographical boundaries [55]. More importantly, the online research method used in this study is appropriate for the research context in OHCs. This study was reviewed and approved by the China Three Gorges University Institutional Review Board. Then we distributed the recruitment announcement to recruit experiment subjects on the university message board. All participants were volunteered and provided written informed consent to participate in the study. In this study, gender, age, and the use of the online healthcare community will be factored into the observation of individual samples’ characteristics. In a sample of 386, 56.5 percent is female, while male accounts for 43.5 percent. Their ages focus on the range from 18 to 35, and about 95 percent of the subjects hold post-school qualifications. The results of the frequency analysis of demographic variables are shown in Table 1.

**Table 1:** Statistical description of the sample

Variables	n	Percent	Mean	Standard Deviation
*Gender*				
Male	168	43.5	1.56	0.496
Female	218	56.5		
*Education*				
Senior high school and below	22	5.7	2.883	0.815
Junior college	78	20.2		
Undergraduate	218	56.5		
Master	59	15.3		
Doctor	9	2.3		
*Age (years)*				
≤ 24	135	35.0	2.16	1.106
25–30	112	29.0		
31–35	95	24.6		
36–40	29	7.5		
> 40	15	3.9		

### Procedures

Our study creates a vignette that places participants in a situation where they would need to seek medical care at an OHCs. Vignettes “present subjects with written descriptions of realistic situations and then request responses on several rating scales that measure the focal dependent variables” [56]. This manipulation aims to provide control by placing all subjects in the same scenario with the only difference being the strength of the argument based on PI. This method has been validated [50] for capturing individual perception and trust development in virtual communities [51, 52]. Additional file 1: Appendix A contains a detailed description of the treatments.

The subjects are allocated to one of three situations. Initially, the subjects must fill out a questionnaire with demographic information. Following the task description, the participants were randomly assigned to one of the potential physicians’ profiles information: claim only, claim/data, claim/data/backing, and one part of the information is used to display doctors’ status capital, while the other part is to present their decisional capital. The subjects evaluated the two dimensions of professional capital; initial trust and consultation intention. We can avoid problems of repeated subjects and low participation by controlling the IP address that allows one participant to participate in one experiment and setting the answering time. This will ensure a high level of internal and external validity of the experiment.

### Independent variables

This study manipulated the strength of argument (i.e., claim only, claim/data, and claim/data/backing) and explored the influence on the perception of professional capital (status capital and decisional capital). Earlier studies have shown that users can deal with the information displayed in an online environment [18], and information originating from offline has no significant effect on the behaviors of patients’ online consultations [57]. Therefore, the emphasis is on changing the strength of the argument (its information constitution) shown on the doctors’ homepage to control the parameter types. By randomly presenting three types of information about claim only, claim/data, and claim/data/backing to the subjects, they can identify and evaluate the doctors’ professional capital.

### Dependent variables

This article has four dependent variables (status capital, decisional capital, initial trust, and consultation intention), measured through the Likert questionnaire using 1 (strongly disagree) to 5 (strongly agree). The status capital and decisional capital items were developed based on the conceptual definition and scales from prior research [2]. Initial trust items were used in the current study to assess individual trust during the first interaction process [45]. Items based on Gong et al. [26] measured for consultation intention are developed to predict patients' selection behaviors [20]. Before data collection, a pilot test was conducted with a separate set of participants, including eleven graduate students and two undergraduate students. They were requested to follow the experiment and review the measurement items to evaluate the constructs, semantics, length, and format of the questionnaire, and the questionnaire design was adjusted in response to their feedback. These items are listed in Additional file 1: Appendix B for four dependent variables of interest.

### Control variables

This study included four control variables that might influence the perception of professional capital. Three were demographic (gender, age, education), and one pertained to use experience (Additional file 2).

## Data analysis and results

### Measurement model

Sample data can meet the requirement of normal distribution and homogeneity of variance (L (1.384) = 1.737, *P* > 0.05). We use SPSS17.0 and Smart PLS to make a reliability analysis that shows the accuracy and precision of the questionnaire. We can evaluate it by checking Cronbach's Alpha values. The values of reliability coefficients, an index used to describe the reliability, are 0 to 1. When it is closer to 1, its reliability is higher. According to the overall reliability coefficients, the Cronbach coefficient after standardization is 0.931 (Table 2), which means having high reliability. Cronbach's Alpha values which are deleted are less than the overall reliability coefficients, which are standardized, while their values are all greater than 0.7 (Table 3). Therefore, content dimensions (status capital, decisional capital, initial trust, and consultation intention) do not need to be adjusted, and our scale has a high level of reliability.

**Table 2:** The overall result of reliability analysis

Cronbach's Alpha	The number of items
0.931	14

**Table 3:** The items’ result of reliability analysis

Construct	Factor loading	Cronbach's Alpha	T-statistic	Rhoda
*Decisional capital*				
DC1	0.788	0.813	29.65	0.788
DC2	0.780		32.53	
DC3	0.776		27.27	
DC4	0.782		32.10	
*Status capital*				
SC1	0.765	0.797	27.26	0.788
SC2	0.777		27.90	
SC3	0.762		28.05	
SC4	0.799		32.20	
*Initial trust*				
IT1	0.802	0.791	32.70	0.767
IT2	0.827		43.20	
IT3	0.847		44.30	
*Consultation intention*				
CI1	0.839	0.806	39.10	0.78
CI2	0.808		32.20	
CI3	0.856		55.96	

The validity was measured to validate the extent to which the concept of interest is accurately represented in the measurement scale, and convergent validity and discriminant validity were evaluated to determine the overall validity of the model. The convergent validity, composite reliability and average variance extracted were all evaluated. As shown in Table 4, composite reliability (CR) is all greater than 0.7, and the average variance extracted is above the recommended minimum of 0.50, ranging from 0.6 to 0.7. This result provides support for convergent validity in the current model. In terms of discriminant validity, the square roots of the average variance extracted between constructs were found to be greater than the correlation across constructs, suggesting discriminant validity.

**Table 4:** The result of validity analysis

Construct	CR	AVE	Correlation matrix			
			CI	DC	IT	SC
CI	0.873	0.697	0.835			
DC	0.863	0.611	0.747**	0.782		
IT	0.865	0.681	0.763**	0.779**	0.825	
SC	0.858	0.602	0.776**	0.755**	0.768**	0.776

**When the confidence level (two-sided) is 0.01, the correlation is significant

*CI* Consultation intention; *DC* Decisional Capital; *IT* Initial Trust; *SC* Status Capital; *CR* Composite Reliability; *AVE* average variance extracted

### Structural model

The Structural Equation Model (SEM) was conducted using AMOS 21.0. Absolute fit index (CMIN/DF, GFI, AGFI, RMSEA) and relative fit index (NFI, IFI, CFI) were calculated to evaluating the degree of model fit. As shown in Table 5, CMIN/DF (1.855) < 3, RMSEA (0.047) < 0.05, while the other indexes are all greater than 0.85, which confirms a good fit of the current model.

**Table 5:** The fit index of confirmative factor

	CMIN/DF	GFI	AGFI	RMSEA	NFI	IFI	CFI
Standard value	< 3	> 0.85	> 0.85	< 0.1	> 0.85	> 0.85	> 0.85
Test value	1.855	0.957	0.933	0.047	0.956	0.979	0.979

H1 and H2 respectively examine the difference in perception of professional capital as the strength of argument grows gradually: Claim only, Claim/Data, Claim/Data/Backing. Analysis of Variance (ANOVAs) was conducted and found a significant mean difference for all dimensions of professional capital across the strength of argument, including in the physicians’ PI (Table 6). There are significant differences in the Mean and Standard Deviation of professional capital (decision capital and status capital) under various physician’s information argumentation levels. The different letters between the treatments indicate significant differences. This result suggests the argument strength of information has a profound influence on the perception of decisional capital and status capital, supporting H1 and H2. In addition, a follow-up post hoc Ryan-Eunoto-Gabriel-Welsch F test was conducted to understand how the specific treatments were different from one another. The results show that the personal information fully composed of Claim/Data/Backing results in a stronger impression of decisional capital and so does the impression of social capital, supporting H1a and H2a. The information including both claim and data has a greater perception of decisional capital and status capital than these information with claim only. This result supports H1b and H2b. Most important, the backing has a stronger impact.

**Table 6:** Results of analysis of variance

	n	DC	SC
Claim	140	14.70^b^ (2.58)	14.61^b^ (2.75)
Claim/data	118	15.12^b^ (2.56)	15.14^b^ (2.71)
Claim/data/baking	128	17.02^a^ (2.53)	16.56^a^ (2.61)
F value		30.31***	18.53***

Ryan-ento-Gabriel-Welsch F, *p* < 0.05

***P < 0.001

H3 states that the impression formation of professional capital would positively impact a patient’s initial trust, and H4 illustrate the initial trust’ impact on the consultation intention. Given the analysis result, it is status capital (t = 6.608, β = 0.416, *p* < 0.001) and decisional capital (t = 7.382, β = 0.465, *p* < 0.001) that are influential in forming an initial trust, and initial trust significantly positive influenced the patients’ consulting selection (t = 23.953, β = 0.286, *p* < 0.001).

H3 and H4 suggest that initial trust mediates the impact of professional capital on the overall consulting intention. In this paper, the Bootstrap test method was used to verify the mediation effect and conducted repeated sampling 5000 times. As shown in Table 7, decisional capital (t = 3.718, β = 0.133, *p* < 0.001) and status capital (t = 3.237, β = 0.119, *p* < 0.001) have significant indirect effects on the consultation intention. Furthermore, decisional capital (t = 4.055, β = 0.245, *p* < 0.001) and status capital (t = 5.972 β = 0.373, *p* < 0.001) has a significant direct impact on the consultation intention. Therefore, this study proves that initial trust partially mediates professional capital and consultation intention.

**Table 7:** The structural model assessment for direct and indirect effects

Effect	Sample Mean	Standard Deviation	Std β	T value
*Direct effect*				
DC → CI	0.248	0.060	0.245	4.055***
DC → IT	0.466	0.063	0.465	7.382***
IT → CI	0.285	0.070	0.286	4.081***
SC → CI	0.369	0.062	0.373	5.972***
SC → IT	0.414	0.063	0.416	6.608***
*Indirect effect*				
DC → IT → CI	0.356	0.051	0.133	3.718***
SC → IT → CI	0.319	0.052	0.119	3.237***

***p < 0.001

### Common method bias

As all the data were collected using an internet experiment, there is a possibility for common method bias (CMB) to influence the result’s reliability. This study first addressed this issue by performing a confirmatory factor analysis [58]. CMB, regarded as a latent variable, needs to be added in the structural equation model (SEM). A model M2, including method factors, is constructed to compare the main fitting indexes of our focus structural model. △GFI = 0.005, △IFI = 0.005, △TLI = 0.013, △RMSEA = 0.009, △SRMR = 0.003. The variation of each fitting index is less than 0.02, indicating that CMB may not be a fundamental problem in the data set.

### Robustness test

To ensure the robustness of our findings, we perform a hierarchical regression analysis. Table 8 presents the estimation results of our model. This first shows a model with control variables in Columns (1), followed by independent variables and mediation variables of interest in Columns (2) and (3). The Adjusted R-squared values rose from 0.032 to 0.679 and were statistically significant. The findings are consistent with previous research results. The doctors’ professional capital perception increases patient’ consulting intention, which is mediated by initial trust. Therefore, the results are robust. The multicollinearity test of the model shows that variance inflation factor (VIF) statistics for the variables in the structural model are less than 5, which means that there is no collinearity problem. The D-W value is near 2, which shows that the model has no autocorrelation. There is no correlation between sample data.

**Table 8:** Parameter estimates of the consulting intention (robust check)

	(1)	(2)	(3)
Constant	− 0.123	− 0.008	0.064
	(− 0.574)	(− 0.066)	(0.512)
Gender	− 0.182*	− 0.001	− 0.015
	(− 2.031)	(− 0.027)	(− 0.282)
Education	0.134*	0.009	− 0.006
	(2.457)	(0.286)	(− 0.198)
Usage count	0.103*	0.026	0.024
	(2.42)	(1.002)	(0.99)
Age	− 0.098*	− 0.036	− 0.037
	(− 2.114)	(− 1.277)	(− 1.397)
Status capital		0.493**	0.374**
		(10.721)	(7.601)
Decisional capital		0.370**	0.239**
		(7.877)	(4.686)
Initial trust			0.284**
			(5.506)
N	386	386	386
R^2^	0.042	0.659	0.685
Adjusted R^2^	0.032	0.654	0.679
F	F (4,381) = 4.137	F (6,379) = 122.253	F (7,378) = 117.226

T statistics in parentheses

*p < 0.05; **p < 0.01

## Discussion

### Principal findings

Prior to the online consultation, most patients in the online healthcare community had little knowledge of these doctors, particularly their service quality and professional level. This paper investigated the effect of the strength of the argument in the physicians’ PI on the initial perception of professional capital when these physicians are unknown to the reader, which has a positive impact on consultation intention to these physicians. This finding is in accordance with prior literature indicating that information on physician’s professional level positively impacts patients’ selection online [14, 15, 59]. In the online healthcare context, the strength of the argument (i.e., claim only, claim/data, claim/data/backing) composed of physicians’ PI positively influences the initial impression of professional capital, suggesting PI on the health platform may be effective for forming initial judgements. In turn, these impression affects the patients’ choice, which is mediated by initial trust. In other words, the perception of professional capital goes higher with the increase in the strength of argument presented in the doctor's information, the patients are more likely to believe them and choose the doctor for online consultation on OHCs. Our research provides initial insights about the impact of strength of argument presented in the doctor's information on the impression formation process of professional capital. Besides, not all dimension of perception capital perceptions is influenced in the same way as the strength of argument increases. The increase of the strength of argument included in the physicians' information has a stronger impact on the impression formation of decisional capital. First, this is because the average title of doctors who participated in the online communities is above the level of associate chief physicians, with 90 percent of them engaged in clinical and research work in the top three hospitals. There is no significant difference in physician title and affiliated hospital, and it has a negligible effect on the perception of status capital. Besides, patients pay more attention to the accuracy and efficiency of physicians' diagnosis in mediated environment.

Perceptions of professional capital (status capital and decisional capital) are highest when a fully composed argument (claim/data/backing) is included in a profile, with claim/data being the next highest and claim only the lowest. Recommendations from others have the strongest impact, rather than the self-generated claim. This result is supported by Walther and Parks [60], which suggests that individuals tend to exaggerate their abilities in an online environment, and information from others (such as electronic word-of-mouth) is more objective, convincing, and trustworthy. Meanwhile, the individual has a greater sense of identification with others who have identical illnesses or similar symptoms, and their recommendations and suggestions are more likely to be accepted. Finally, initial trust mediated the effect of perception of professional capital on the patients’ consultation intention online. According to the personal information on the physicians’ homepage, patients make an initial judgment about their status and capacity. Patients are willing to believe that doctors with higher status and decision-making ability are more helpful and, therefore, more likely to choose these physicians. This result is inconsistent with the previous study, which suggests the perception of professional capital and trust are developed over time through ongoing interaction [61, 62]. However, our research shows that perceptions of professional capital can develop before individuals interact.

### Limitation and future research directions

The limitations of this study are typical of experimental research. This experiment is being conducted online to recruit participants and collect data. In the online medical community, the scene-based vignette replaces the actual situation. Some researchers continue to have reservations about the online experiment, believing that the people participating in it are not representative. There are issues with reoccurring subjects and low participation. On the other hand, this study focuses on the effect of information included on physicians' homepages on patients' intention to consult in OHCs. It is the subjects who are groups who become acquainted with the network. As a result, it is thought that recruiting participants via the internet is more representative. In addition, the time and IP address control for answering questions can effectively avoid repeated subjects and low participation questions.

This research brings a fresh perspective to OHCs and focuses on the impression formation of professional capital before physician–patient interactions. The contents of the profiles presented in this study were all positive, which is typical of research using the Toulmin’s model. Future research needs to examine the impact of negative information presented in the profiles on patients’ information evaluation. In addition, future research should further examine the differences between patients and physicians to understand information processing better.

### Implication for research

Many physicians provide medical services in OHCs, whose competence and service quality are uneven. Due to the limitation of information, it is difficult for patients to evaluate the physicians’ service quality and competence and decide which one to consult after visiting the physicians’ homepage. The poor choice often reduces the efficiency and effectiveness of diagnosis and treatment. To evaluate a physician’s medical quality and professional competence, patients are more likely to take full advantage of various kinds of information to reduce uncertain risks, including system-generated and self-generated information. Is it a meaningful way to browse PI to know physicians in the health communities? To what extent does the creation of strong relationships in a virtual environment depend on the perception of professional capital (perhaps better assessed using PI)? We need more theory and research to understand how and why patients’ selection online so we can better understand the role that PI should play. As a result, this paper examines (1) the impact of physicians’ PI appearing in OHCs on the impression of physicians’ professional capital, (2) whether these pieces of information are equal value to patients, and (3) how these first impressions influence patients’ choice online. Our study makes several contributions to theoretical implication.

The extensive studies on the use of IT and UGC in healthcare have mainly examined the potential impact of demand-side physician reviews (such as online word-of-mouth, online rating) on patients’ recommendations [13], online-offline behavior [3], and online selection [63]. However, we have little theory or research on the effect of doctors’ PI from the supply side. Furthermore, these previous studies have investigated the effect of patient-generated or system-generated information, respectively. In contrast, there is a lack of adequate literature about the effects of various types of information on patient decisions regarding online healthcare services. Since medical services are intangible and heterogeneous, it is more difficult for patients to evaluate service quality than other services. Therefore, to more accurately evaluate the physicians’ professional level, patients would comprehensively consider all kinds of information to reduce uncertainty. As a result, this research investigates the impact of doctor-provided information and system-generated information on online patient selection. Our research adds to this literature and provides new empirical evidence in this critical area.

Some authors have long recognized the trichotomy of products and services search, experience, and credence qualities [64]. Prior research on search and experience goods generally finds that PI of experience service is effective [65]. However, our study extends this insight to credence goods. Our paper primarily focuses on the role of PI in credence goods with chronic disease care. Our study presents some implications for theory and research to link the different components of PI of experience to patients’ self-selection behavior online.

Although there is plenty of theory or research on information sharing behavior and online doctor-patient interaction on patient satisfaction during the online physician service delivery process [66, 67]. According to our findings, the strength of argument demonstrated has a significant impact on patients' initial impressions of professional quality and social status as they begin to search on the physicians' personal websites without individual interaction. These impressions frequently serve as the foundation for future physician–patient interactions online. More theory and research on impression formation on healthcare websites is thought to be required as a result.

Minimal research has been conducted within OHCs to examine the model of argumentation (Toulmin' Model of Argumentation). This study extends the argumentation model by showing that the strength of the argument is significant in medical websites; specifically, personal information is classified into different strengths (e.g., claim, data, backing) and has various values. Recommendations from patients with similar illnesses had a stronger effect on the initial impression of decisional capital. Results suggest that the argumentation model provides a framework for understanding the perception formation process when information is provided online.

Finally, the existing research on professional capital shows that the perception of status capital and decisional capital only can develop through continuous interaction. However, this study suggests that it can be pre-presumed and built based on the information before in-person interactive online. Much like trust, the research believes trust can be developed over time. Nevertheless, research in a virtual environment demonstrated that trust was often granted ex -ante or presumptively before interaction [61]. More professional capital theory and research are needed to better understand the extent to which professional capital is developed over time through personal interactions versus granted ex-ante based on physician profiles.

### Implication for practice

The results from this paper have several significant practical implications for online healthcare websites, physicians, and patients. The various types of physician information presented on these websites can greatly determine how patients perceive physicians on their status and professional ability, which will affect patients' initial trust and consulting intention. Results demonstrate that patients use various components included in the PI to perceive physicians’ ability before consultation and interact online. Mostly, not all information is of the same importance.

From OHCs’ perspective, platform providers can better understand how patients translate these informational signals and separate high-expertise doctors from low-expertise doctors. In addition, results suggest that these information compositions are not equal value to patients [68]. Patients are subject to believe system-generated information rather than self-generated by physicians themselves. As a result, medical platforms should develop precise algorithms to recommend and provide statistical information about physicians' competence and service quality. This provides a credible endorsement for physicians' claims and assists patients in reducing perceived uncertainty to form an initial impression of physicians' professional capital. In addition, the platform will more strictly review the authenticity of doctors' personal information When registering to reduce the degree of self-manipulation. Besides, this study illustrated how are perceptions of professional capital influenced by the overall PI design (e.g., the inclusion of specific components). Platform providers should allow physicians to customize profiles to position various information in different places in the profile.

Additionally, the overall design and layout of PI could be changed to emphasize certain characteristics that would be helpful in the health communities. For example, the platform provider may emphasize recommendations to increase the perception of professional capital and develop trust. This will provide an opportunity for patients to better understand their target physician.

Most physicians can use impression management to enhance their professional image and tailor their online representation more effectively in the mediated environment. Especially, they should attach importance to the design of their homepage from status and decision-making aspects, especially about the recommendation from others. By focusing on professional competence, physicians can attract new patients and gain a wealth of clinical experience, allowing them to achieve economic and social returns. Therefore, physicians could provide better service to these patients and make a better impression when potential patients review interactions between the doctors and their current or previous patients. Furthermore, it allows some capable but unknown doctors to become well-known.

From the patient perspective, PI on the websites can reduce information asymmetry, perceived risk, and lack of trust that currently plagues the virtual environment. This study illustrates that viewing the physicians’ homepage can help form initial professional impressions that may attribute to building trust relationships during early interactions as well as learning more about physicians. The patient should take full advantage of PI to learn more about these doctors and select the most experienced one to consult. They should not simply rely on online physician reviews or other demand-side information provided but also consider PI.

## Conclusion

An increasing number of physicians from various hospitals provide medical services in OHCs. Patients can find a wealth of information about physicians displayed on their homepage and visit a physician after browsing this information. Therefore, this study focuses on patients' online selection of a physician from the standpoint of impression formation. According to the findings, the difference in argument strength displayed on the physicians' homepage has a significant impact on the perception of professional capital. Through initial trust, perception influences a patient's selection behavior indirectly.

## Supplementary Information

**Additional file 1. Appendix A**. Detailed Description of Experiment Manipulations.** Appendix B**. Measurement Items.

**Additional file 2**. The experiment data collected.

## Data Availability

All data generated and analyzed during this study are included in this published article and its Additional files.

## Abbreviations

OHCs: Online health communities
CMB: Common method bias
UGC: User-generated content
IT: Information technology
PI: Profile Information

## Footnotes

1 The 49th Statistical Report on China’s Internet Development (http://www.cnnic.net.cn).
