# Supplementary material for: Online selection of a physician by patients: the impression formation perspective
Source: BMC Med Inform Decis Mak. 2022 Jul 25;22:193. doi: 10.1186/s12911-022-01936-0 (PMC9309235; doi:10.1186/s12911-022-01936-0)
Supplement: Supplementary file 1 — Additional file 1. Appendix A. Detailed Description of Experiment Manipulations. Appendix B. Measurement Items. [file 12911_2022_1936_MOESM1_ESM.docx]

**Appendix A Detailed Description of Experiment Manipulations**

**Step 1: Sample Vignette Design**

Imagine you are in the following situation:

Website Y is a Chinese online healthcare community with over 820 thousand physicians. Website Y wants patients to get to know these physicians better by allowing them to create profiles.

You are sick today and browsed the website Y. Will you choose this physician to consult on the website Y after reviewing his or her profile information?

**Step 2: Experiment Procedure**

Prior research has found that gender, age and photos may impact a person’s perception of personal profiles information in the virtual environment [1, 2]. Therefore, the same profile picture, including gender and appearance, was chosen for all subjects to minimize any bias based on the physician’s characteristics and test the argument strength of profile information. Besides, the ordering changes randomly to limit any potential confusion in the display order of profile information. For example, one subject first sees the information showing the doctor's status capital (Character A), then the information about decisional capital (Character B), while another participant may see Character B firstly and then Character A. A detailed description of the experimental treatments is provided in Table A1.

Toulmin's argumentation model was used to determine the strength of the argument represented by the PI. Specific elements and information related to each dimension of professional capital assessed are included in each physician PI displayed. For example, a profile for status capital included a claim under the profile summary. "I am a chief physician in a top hospital and have been engaged in clinical, scientific research, and teaching work for many years," it added. Data is represented by information provided by physicians to support this summary statement. In this study, we collected data from a variety of sources, including clinic title, academic title, and affiliated hospital level. On the PI, the backing is presented as "Title of Good Doctor of The Year." The presentation of information that could elicit perceptions of professional capital that was not being assessed was controlled. Table A2 contains a complete list of the manipulated profile information.

Table A1 Experimental Treatments

| Profile Information | Argument Strength | Status Capital | Decisional Capital |
| --- | --- | --- | --- |
| Self-Generated | Claim Only | Character A1 – Status Capital Claim | Character B1 – Decisional Capital Claim |
|  | Claim+Data | Character A1 – Status Capital Claim  Character A2 – Status Capital Data | Character B1 – Decisional Capital Claim  Character B2 – Decisional Capital Data |
| System-Generated | Claim+Data+Backing | Character A1 – Status Capital Claim  Character A2 – Status Capital Data  Character A3 – Status Capital Backing | Character B1 – Decisional Capital Claim  Character B2 – Decisional Capital Data  Character B3 – Decisional Capital Backing |

Table A2 Profile Information Manipulated across Professional Capital

| Dimension | Claim | Data | Backing |
| --- | --- | --- | --- |
| Status Capital | Profile summary statement | Doctor’s Clinic Title,  Doctor’s Academic Title,  Affiliated Hospital Level, | Title of Good Doctor of The Year, |
| Decisional Capital | Profile summary statement | Quantity of Consultation,  Quantity of Medical Articles，  Quantity of National Fund Projects， | Recommendation,  Quality Rating,  Quantity of virtual gift, |

**Appendix B Measurement Items**

| Decisional Capital[3, 4] | |
| --- | --- |
| DC1 | I feel that this physician is so enthusiastic and dedicated that he/she is willing to interact dynamically with patients. |
| DC2 | I feel that this physician has rich experience in terms of clinical judgment. |
| DC3 | I feel that this physician makes a medical judgment based on the evidence and clinical experience |
| DC4 | I feel that this physician makes the precise medical judgment following the profession commitment |
| Scale of items: 1=strongly disagree to 5=strongly agree | |
| Status Capital[3, 4] | |
| SC1 | I feel that this physician is at a higher level among social professionals |
| SC2 | I feel that this physician is entitled to higher priority and privileges so that has higher medical resources. |
| SC3 | If needed, I believe that this physician can take advantage of his medical resource to assist the patient to recuperate faster and more effectively |
| SC4 | I feel that this physician can have access to personal and social advantages of the structural power of social professionals and use it to make an accurate judgment |
| Scale of items: 1=strongly disagree to 5=strongly agree | |
| Initial Trust[5] | |
| IT1 | I believe that this physician is necessarily medically qualified |
| IT2 | I believe that the diagnosis and treatment provided by this physician are reliable |
| IT3 | I believe that this physician is trustworthy |
| Scale of items: 1=strongly disagree to 5=strongly agree | |
| Consultation Intention[6] | |
| CI1 | I will consult this physician in the future |
| CI2 | I predict that I will consult health issues with this physician when needed in the future |
| CI3 | If I have chance to consult this physician, I will choose him/her |
| Scale of items: 1=strongly disagree to 5=strongly agree | |

### References

1. Walther, J.B., et al., The Role of Friends’ Appearance and Behavior on Evaluations of Individuals on Facebook: Are We Known by the Company We Keep? Human Communication Research, 2008. 34(1): p. 28-49.

2. ter Stal, S., et al., Who Do You Prefer? The Effect of Age, Gender and Role on Users’ First Impressions of Embodied Conversational Agents in eHealth. International Journal of Human–Computer Interaction, 2019. 36(9): p. 881-892.

3. Guo, S., et al., How Doctors Gain Social and Economic Returns in Online Health-Care Communities: A Professional Capital Perspective. Journal of Management Information Systems, 2017. 34(2): p. 487-519.

4. Liu, X., et al., How to Manage Diversity and Enhance Team Performance: Evidence from Online Doctor Teams in China. Int J Environ Res Public Health, 2019. 17(1).

5. Gao, L. and K.A. Waechter, Examining the role of initial trust in user adoption of mobile payment services: an empirical investigation. Information Systems Frontiers, 2015. 19(3): p. 525-548.

6. Cao, X., et al., Online selection of a physician by patients: Empirical study from elaboration likelihood perspective. Computers in Human Behavior, 2017. 73: p. 403-412.
